# Automated detection of the contrast phase in MDCT by an artificial neural network improves the accuracy of opportunistic bone mineral density measurements

**DOI:** 10.1007/s00330-021-08284-z

**Published:** 2021-10-23

**Authors:** Sebastian Rühling, Fernando Navarro, Anjany Sekuboyina, Malek El Husseini, Thomas Baum, Bjoern Menze, Rickmer Braren, Claus Zimmer, Jan S. Kirschke

**Affiliations:** 1 Department of Neuroradiology, School of Medicine, Klinikum rechts der Isar, Technical University of Munich, Ismaninger Str 22, 81675 Munich, Germany; 2 Department of Informatics, Technical University of Munich, Munich, Germany; 3 Center for Translational Cancer Research (TranslaTUM), Klinikum rechts der Isar, Munich, Germany; 4 Department of Radio Oncology and Radiation Therapy, Klinikum rechts der Isar, Munich, Germany; 5 Department of Quantitative Biomedicine, University of Zurich, Zurich, Switzerland; 6 Department of Diagnostic and Interventional Radiology, School of Medicine, Klinikum rechts der Isar, Technical University of Munich, Munich, Germany

**Keywords:** Bone density, Osteoporosis, Multidetector computed tomography, Machine learning, Screening

## Abstract

**Objectives:**

To determine the accuracy of an artificial neural network (ANN) for fully automated detection of the presence and phase of iodinated contrast agent in routine abdominal multidetector computed tomography (MDCT) scans and evaluate the effect of contrast correction for osteoporosis screening.

**Methods:**

This HIPPA-compliant study retrospectively included 579 MDCT scans in 193 patients (62.4 ± 14.6 years, 48 women). Three different ANN models (2D DenseNet with random slice selection, 2D DenseNet with anatomy-guided slice selection, 3D DenseNet) were trained in 462 MDCT scans of 154 patients (threefold cross-validation), who underwent triphasic CT. All ANN models were tested in 117 unseen triphasic scans of 39 patients, as well as in a public MDCT dataset containing 311 patients. In the triphasic test scans, trabecular volumetric bone mineral density (BMD) was calculated using a fully automated pipeline. Root-mean-square errors (RMSE) of BMD measurements with and without correction for contrast application were calculated in comparison to nonenhanced (NE) scans.

**Results:**

The 2D DenseNet with anatomy-guided slice selection outperformed the competing models and achieved an F1 score of 0.98 and an accuracy of 98.3% in the test set (public dataset: F1 score 0.93; accuracy 94.2%). Application of contrast agent resulted in significant BMD biases (all *p* < .001; portal-venous (PV): RMSE 18.7 mg/ml, mean difference 17.5 mg/ml; arterial (AR): RMSE 6.92 mg/ml, mean difference 5.68 mg/ml). After the fully automated correction, this bias was no longer significant (*p* > .05; PV: RMSE 9.45 mg/ml, mean difference 1.28 mg/ml; AR: RMSE 3.98 mg/ml, mean difference 0.94 mg/ml).

**Conclusion:**

Automatic detection of the contrast phase in multicenter CT data was achieved with high accuracy, minimizing the contrast-induced error in BMD measurements.

**Key Points:**

*• A 2D DenseNet with anatomy-guided slice selection achieved an F1 score of 0.98 and an accuracy of 98.3% in the test set. In a public dataset, an F1 score of 0.93 and an accuracy of 94.2% were obtained.*

*• Automated adjustment for contrast injection improved the accuracy of lumbar bone mineral density measurements (RMSE 18.7 mg/ml vs. 9.45 mg/ml respectively, in the portal-venous phase).*

*• An artificial neural network can reliably reveal the presence and phase of iodinated contrast agent in multidetector CT scans (* *https://github.com/ferchonavarro/anatomy_guided_contrast_c* *). This allows minimizing the contrast-induced error in opportunistic bone mineral density measurements.*

## Introduction

Abdominal multidetector computed tomography (MDCT) is a widely used method to evaluate a broad range of pathologies [1]. In the USA alone, more than 91 million CT scans were performed in 2019, compared with around 35 million CT scans in 2000 (i.e., 278.5 vs. 123.7 scans, respectively, per 1000 inhabitants) [2]. Besides visual and anatomical information, each CT scan contains extensive biometric data [3]. This potentially useful information could add value to every examination and help address the increasing socioeconomic burden and demands on imaging services worldwide. To date, however, this data mostly remains unused [4].

Over the recent years, advances in computational performance, data processing, and the availability of large datasets have promoted the application of artificial intelligence [5]. In particular, CT imaging has been intensively studied for the application of deep-learning algorithms [6–8]. These frameworks potentially enable fully automated biomarker extraction independent of the clinical indication for CT imaging, commonly referred to as opportunistic screening. In fact, several studies have already shown the benefits of automated and semi-automated extraction of tissue biomarkers, most notably in the field of osteoporosis (i.e., extraction of bone mineral density (BMD), fracture detection, and prediction of fracture risk) [9–11].

Many technical factors can influence the accuracy and precision (reproducibility) of opportunistic CT measurements. Scanner-specific factors include scanner type, tube voltage, and reconstruction kernel. Additionally, the application of iodinated contrast agent in a majority of CT scans also results in a significant bias in Hounsfield unit (HU) attenuation for various tissues [12, 13]. For spinal bone measurements, for example, means of BMD estimates may increase up to 13% on portal-venous (PV) scans [14]. It follows that measurements in contrast-enhanced CT scans should be adjusted to avoid possible misdiagnoses, such as of osteoporosis [15]. Although modern CT scanners usually provide information on contrast administration in the imaging metadata, there is commonly no direct documentation on the contrast phase present and any application errors that may have occurred [16]. Furthermore, these metadata are often incompletely reported, causing major problems for fully automated pipelines.

Thus, the purpose of this paper was (1) to introduce a framework based on an artificial neural network (ANN) that automatically detects the presence and phase of iodinated contrast in an abdominal CT scan and (2) to assess the error in BMD calculation with vs. without such an automated correction.

## Methods

The local institutional review board approved this HIPPA-compliant retrospective study and waived written informed consent (waiver number: 27-19S-SR; 22.04.2020).

### Study population and datasets

**Fig. 1:**
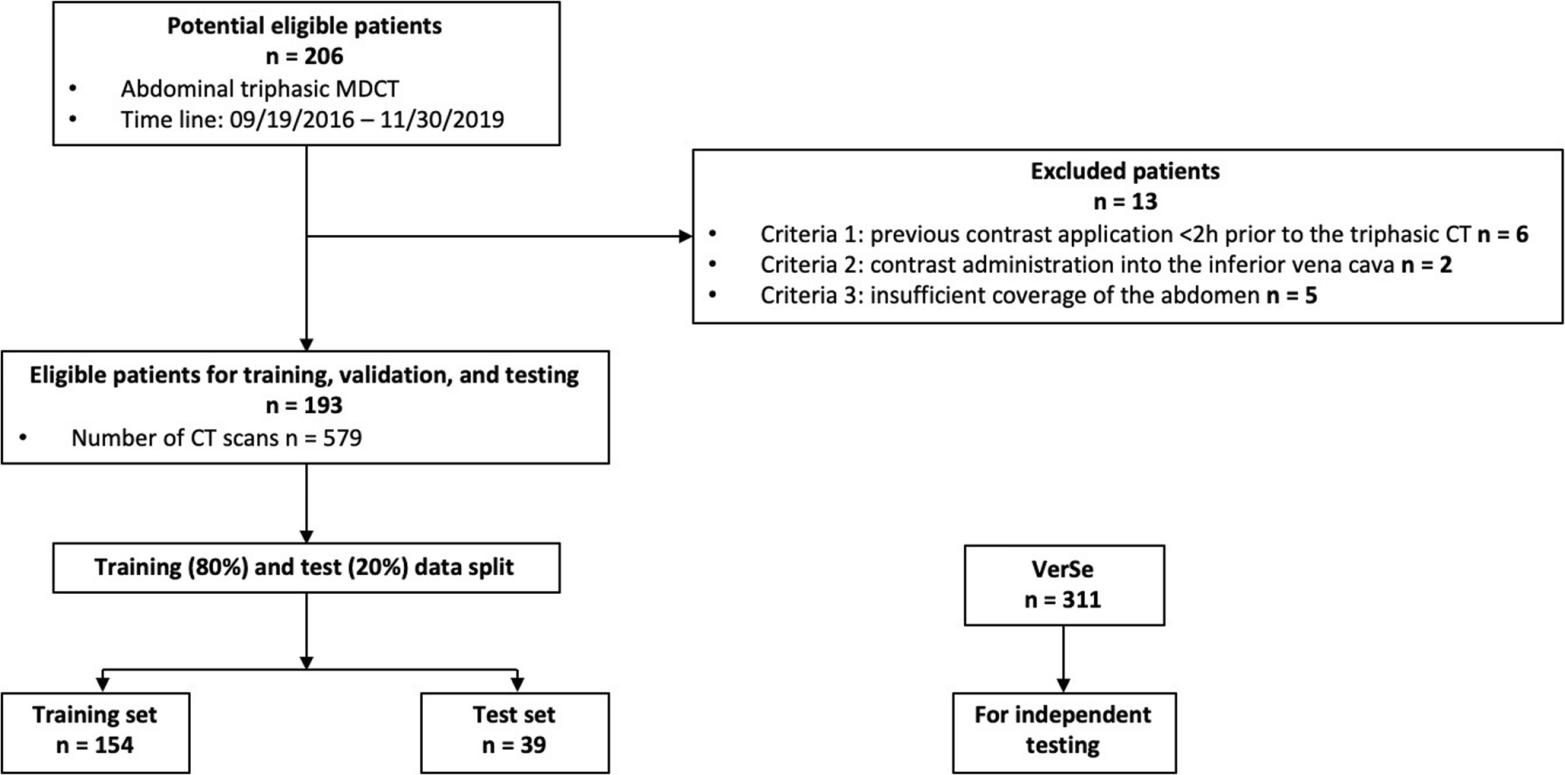
The flowchart shows the data collection process. In total, 193 patients and 579 scans were collected for the study set. This dataset was split into training and test sets. Additionally, another public dataset (VerSe) with 311 patients was included for independent testing

CT images were retrospectively selected from our digital picture archiving communication system (PACS) (Sectra AB). We included 206 consecutive patients with a routine abdominal triphasic MDCT scan (dedicated to investigating liver or kidney pathologies) acquired between September 2016 and November 2019. Exclusion criteria were previous contrast application < 2 h prior to the triphasic CT (*n* = 6), contrast administration via the inferior vena cava (*n* = 2), and insufficient coverage of the abdomen (*n* = 5). The final dataset consisted of 193 adults (48 woman and 145 men), with a mean age of 62.4 ± 14.6 years (Table 1 and Fig. 1). Most patients included were suspected or proven to have liver or kidney cancer (higher male-to-female ratio), resulting in more males being included in the study population. We randomly split the study set into 80% for training (154 patients, 462 scans, 1456 vertebrae) and 20% for testing (39 patients, 117 scans, 411 vertebrae). The split was held consistent during our study. The training set was used to train the different ANN models using a threefold cross-validation. The test set was used to evaluate the different ANNs in unseen CT scans. A public MDCT dataset, VerSe (https://osf.io/nqjyw/;https://osf.io/t98fz/; CC BY-SA), was used to further evaluate the generalizability of our approach [17–19]. We selected all scans that contained at least two vertebrae between the 10th thoracic vertebra and the 4th lumbar vertebra, resulting in 311 patients (158 women and 153 men) with a mean age of 59.6 ± 17.2 years.

**Table 1:** Characteristics of CT scans and patients in the different datasets

	Study set			Public dataset Verse
	Training	Test	All	All
Patients				
No. of patients	154	39	193	311
No. of women	42	6	48	158
Age †	61.9 ± 14.5	62.1 ± 15.2	62.4 ± 14.6	59.6 ± 17.2
Imaging				
No. of scans	462	117	579	311
No. of vertebrae	1456	411	1867	3953
No. of fractures*	30	9	39	N/A
Intravenous contrast				
Nonenhanced	154	39	193	152
Arterial phase	154	39	193	28
Portal-venous phase	154	39	193	131
Scanner				
Philips IQon	154	39	193	86
Philips Brilliance 64	0	0	0	50
Philips iCT	0	0	0	38
Siemens Definitions AS+	0	0	0	38
Siemens Definition AS	0	0	0	53
Siemens Biograph 64	0	0	0	9
Siemens Sensation Cardiac	0	0	0	3
Other	0	0	0	67

Note: Unless otherwise indicated, data are numbers of patients

^*^Only fractures at vertebral level L1–L3 were excluded from BMD assessment

^†^Data are means ± standard deviations

### CT imaging

In the study set, all CT scans were performed on the same MDCT scanner (IQon Spectral CT; Philips Medical Care) using a standardized protocol. The routine abdominal contrast-enhanced images were acquired in a helical mode with a peak tube voltage of 120 kVp, an axial slice thickness of 0.9–1 mm, and an adaptive tube load. After the acquisition of pre-contrast images, all patients received standardized intravenous administration of contrast agent (Iomeron 400; Bracco) using a high-pressure injector (Fresenius Pilot C; Fresenius Kabi). Thirty-seven patients additionally received oral contrast (Barilux Scan; Sanochemia Diagnostics). Post-contrast scans were performed in both AR and PV phases. The acquisition of the AR contrast phase was triggered after a threshold of 120 HU was reached in a region of interest (ROI) placed in the aorta. The PV phase was performed after a standard delay of 80 s. For further analysis of the study, reformations of the spine were reconstructed using a filtered back projection favoring sharpness over noise (bone kernel). In the public dataset VerSe, CT scans were acquired with more than 7 different scanners from different vendors (Table 1) [19]. Here, the contrast phase was visually assessed by two radiologists (2 and 19 years of clinical experience) and served as ground truth. The CT data were converted into the Neuroimaging Informatics Technology Initiative (NIfTI) format and reduced to a maximum of 1 mm isotropic spatial resolution.

### Vertebrae localization, labelling, and segmentation

An offline version of the freely available web tool Anduin (https://anduin.bonescreen.de) was used for fully automated spine processing [18]. Here, a low-spatial-resolution 3D ANN created Gaussian heat maps and extracted bounding boxes around the spine, allowing the extraction of localized maximum-intensity projections (MIPs) to locate the spine. Second, a 2D Btrfly Net was applied on the coronal and sagittal MIPs for vertebra labeling [20, 21]. The correct labeling of the vertebrae was verified by a radiologist and manually corrected if needed. Third, segmentation masks were created around vertebral labels using a 3D U-Net [22, 23]. Fourth, another 3D U-Net was used to divide segmentations in vertebral subregions, including posterior elements as well as the cortical shell and trabecular compartment of the vertebral bodies.

### Data preprocessing

Three different ANN models (2D random DenseNet, 2D anatomy-guided DenseNet, and 3D DenseNet) were explored for our contrast prediction framework. For the 2D models, all volumes were resampled to an isotropic resolution of 1 mm^3^ and normalized using *z*-score normalization. Restricted by the pre-trained architecture for the 2D random models, crop-padding to an image size of 224 × 224 was applied. Due to GPU memory constraints, for the 3D model, all scans were resampled to 3 mm^3^ isotropic resolution and normalized using *z*-core normalization.

### Training of the artificial neural network models

All ANN models were developed in PyTorch (version 1.7.0, https://pytorch.org) using a 48-GB Nvidia RTX 8000 [24]. 2D models were trained with a batch size of 100 and a learning rate of 1e^−4^ using an Adam with weight decay (AdamW) optimizer. 3D models were trained with a batch size of 32 and a learning rate of 4e^−4^. Training was performed with early stopping and monitoring of the validation F1 score to select the best model. Categorical weighted cross-entropy was used as the loss function. Heavy data augmentation was applied at training time and included vertical and horizontal flip, random rotation, random zoom, random cropping, and random field of view. A threefold cross-validation was performed when training the different ANN models. Here, we randomly split the 154 patients (462 scans) from the training set into 3 consecutive subsets (folds). A random seed was set to achieve reproducibility of the training results. During cross-validation, one of the folds was used as the validation set, and the other two folds were used for training. This process was repeated three times, always leaving one different fold for validation. The final accuracy and the best model were detected by tracking the F1 score. Finally, after the optimization, each ANN model was tested in unseen CT scans in the test set and in the public dataset VerSe.

### Characteristics of the different ANN models

**Fig. 2:**
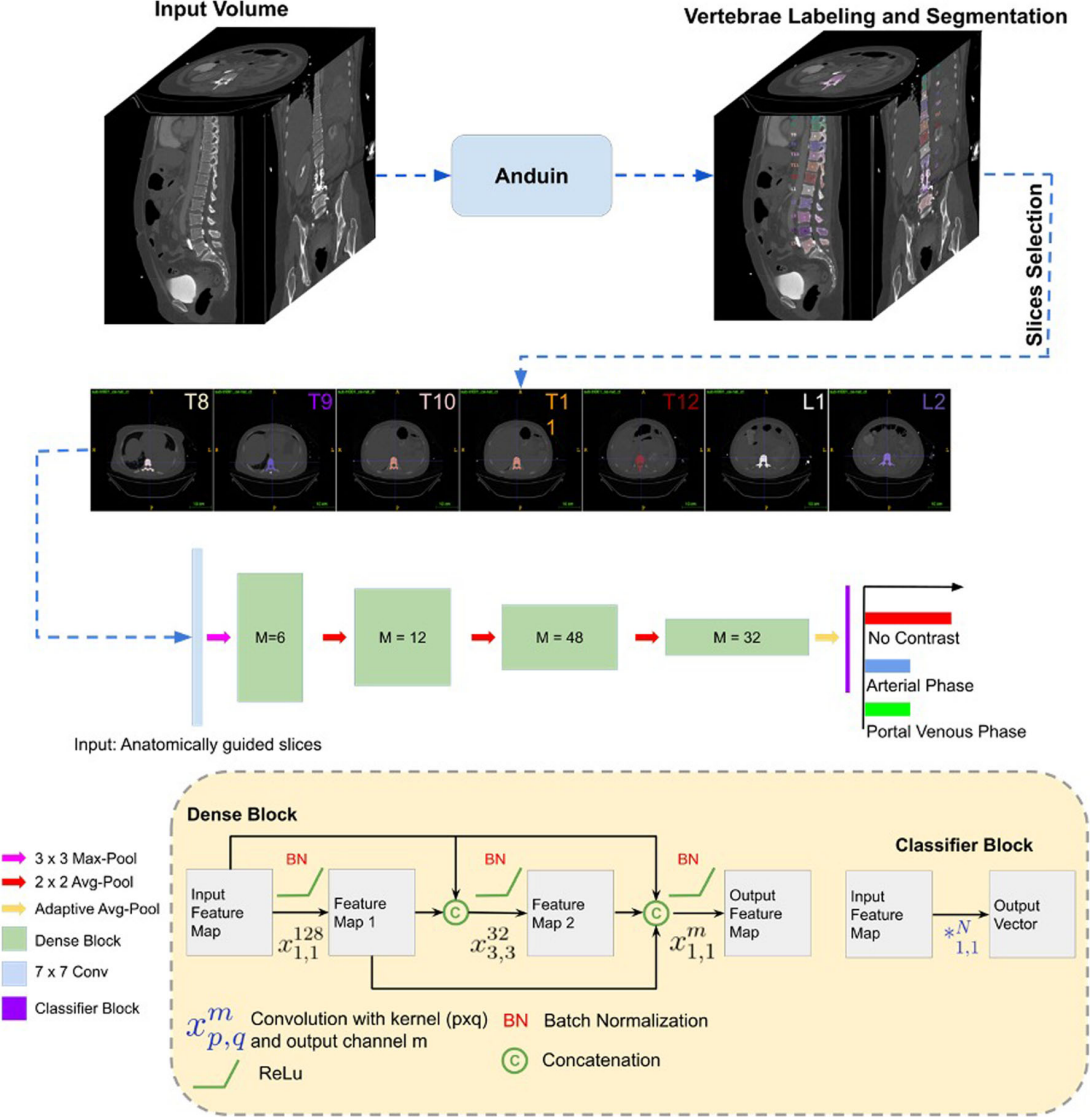
Overview of the fully automated contrast prediction pipeline. First, Anduin (https://anduin.bonescreen.de) is used to localize, label, and segment the vertebrae. Second, the 2D anatomy-guided DenseNet selectively extracts axial slices from the CT scans based on vertebral centroids T8–T12 and L1–L2. These seven images serve as the patient-specific input for a DenseNet161 network depicted in the bottom panel of the figure. The network generates seven contrast predictions, one for each image. The average of these predictions is calculated, and the contrast phase with the highest value is displayed as the final prediction

Three different ANN models (2D random DenseNet, 2D anatomy-guided DenseNet, and 3D DenseNet) were explored for our contrast prediction framework. The anatomy-guided model (2D anatomy-guided DenseNet, https://github.com/ferchonavarro/anatomy_guided_contrast_ct) selectively extracted axial slices from the CT scans based on vertebral centroids that were obtained with the automated pipeline Anduin (Fig. 2). Here, we evaluated different combinations of thoracic and lumbar vertebrae levels. The anatomy-guided model that combined axial images from T8, T9, T10, T11, T12, L1, and L2 achieved the best performance in the validation sets. The axial images at those different spine levels served as input to the ANN, resulting in a probability vector for each image for each contrast phase (AR, NE, PV). The final contrast prediction was determined by majority vote from all available predictions in a specific scan. The naive random slice selection model (Random 2D) randomly used seven axial slices independent of the vertebral centroids. The final contrast prediction for this model was calculated similarly to the anatomic-guided model. For both the anatomic-guided model and the 2D random model, a pre-trained DenseNet161 was used as the deep-learning model architecture [25]. The 3D model (3D DenseNet) used the full 3D scan as input [26].

### Fracture evaluation and BMD extraction

In the test set, CT scans were screened for fractures using a semiquantitative approach according to Genant [27]. Vertebrae were graded into non-fractured (grade 0) and fractured according to height loss (grade 1, 20–25%; grade 2, 25–40%; and grade 3, ≥ 40%). Abnormal morphometry related to developmental changes, like in Scheuermann disease, was not rated as a fracture. Vertebrae at the levels of L1–L3 that had a fracture grade greater than 1 were excluded from further BMD assessment (*n* = 22). BMD values were automatically extracted from the segmentations masks of the trabecular compartment of vertebral bodies, and scanner-specific HU-to-BMD conversion equations previously calculated with density reference phantoms (QRM) were applied [28]. BMD values were averaged over non-fractured lumbar vertebrae L1–L3 and linear correction equations calculated in the training set were applied for each contrast phase.

### Statistical analysis

Statistical analyses were performed by using Prism 8 (Version 9.0.0, 2020, GraphPad Software). BMD values derived from contrast-enhanced (AR and PV) scans were directly compared with BMD values derived from NE scans using root-mean-square errors [29]. Mean errors and 95% confidential intervals were displayed using standard Bland-Altman plots. Mean BMD values were compared using paired samples *t* test. Statistical significance was defined as *p* < .05. ANN evaluation metrics were obtained using Scikit-learn (version 0.24.2, https://scikit-learn.org/stable/index.html).

## Results

### Automated contrast prediction

**Fig. 3:**
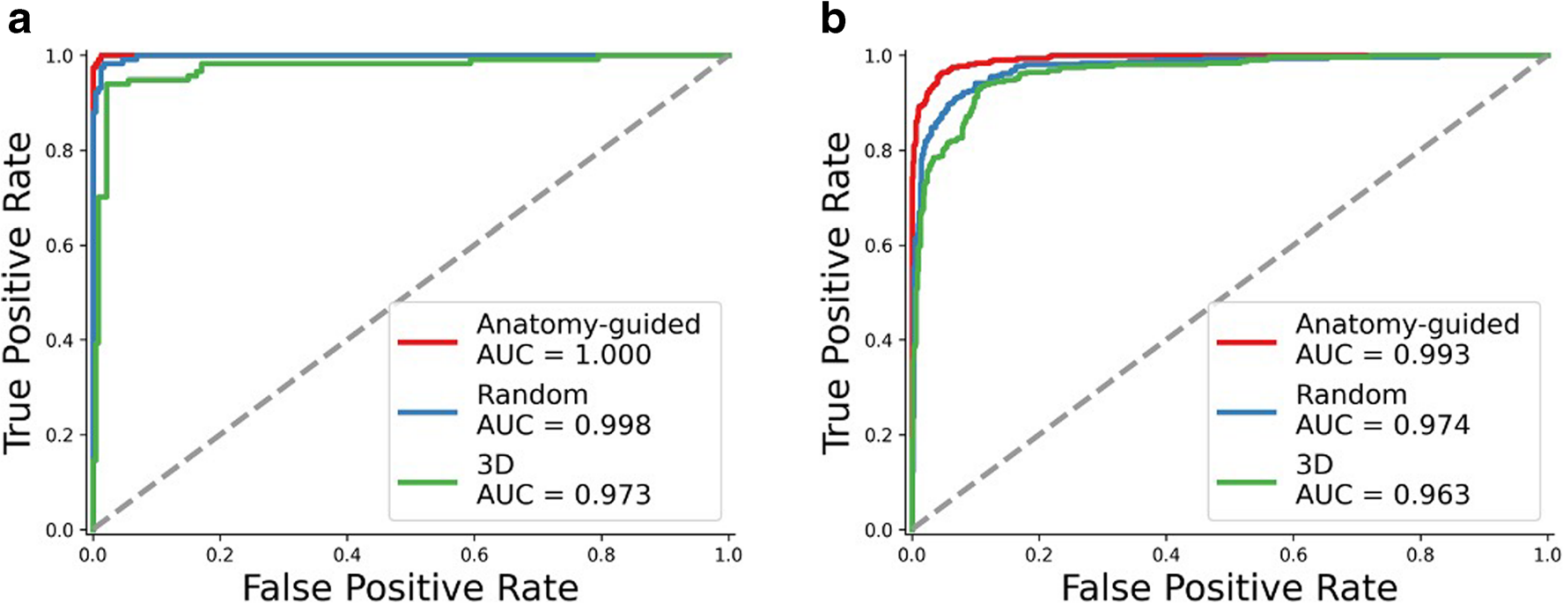
Receiver operating characteristic curves (ROCs) for the different ANN models in both the triphasic MDCT test set (**a**) and the public dataset VerSe (**b**). Red plot: anatomy-guided model; blue plot: 2D random model; green plot: 3D model. AUC = area under the ROC curve

The performance for the different ANN models in the two datasets is tabulated in Table 2 and Table 3. In the study test set, the anatomy-guided 2D DenseNet model achieved the highest F1 score of 0.98 compared to the random 2D DenseNet model (F1 score 0.97) and the 3D DenseNet model (F1 score 0.94). Accordingly, the 2D anatomy-guided approach achieved the best performance in other reported metrics such as precision, sensitivity, specificity, and accuracy (Table 2). Table 3 shows the calculated metrics for the independent public dataset VerSe. Here, among all proposed models, the 2D anatomy-guided approach achieved the best accuracy of 94.2% and an F1 score of 0.93. The random 2D DenseNet model achieved an accuracy of 89% and an F1 score of 0.83. The 3D DenseNet model achieved an accuracy of 84.2% and an F1 score of 0.82. Again, the 2D anatomy-guided approach achieved the highest performance for all other metrics. To further investigate the sensitivity and specificity of our ANN models, we plotted receiver operating characteristic (ROC) curves. Although the area under the ROC curve (AUC) was high (> 0.9) for all approaches (Fig. 3), the 2D anatomy-guided model outperformed all other models in the study test set (AUC 1.00 vs. 0.99 vs. 0.97), as well as in the public dataset VerSe (AUC 0.99 vs. 0.97 vs 0.96) (Fig. 3).

**Table 2:** Evaluation metrics of the different ANNs in the triphasic MDCT dataset

Model	Precision	Sensitivity	Specificity	F1 score	Accuracy
3D	0.941	0.940	0.970	0.940	0.940
Random 2D	0.976	0.974	0.987	0.974	0.974
Anatomy-guided	0.984	0.983	0.991	0.983	0.983

**Table 3:** Evaluation metrics of the different ANNs in the public dataset VerSe

Model	Precision	Sensitivity	Specificity	F1 score	Accuracy
3D	0.827	0.827	0.908	0.827	0.842
Random 2D	0.81	0.86	0.94	0.83	0.89
Anatomy guided	0.946	0.917	0.966	0.931	0.942

### Accuracy errors in BMD measurements before and after the correction for contrast injection

**Fig. 4:**
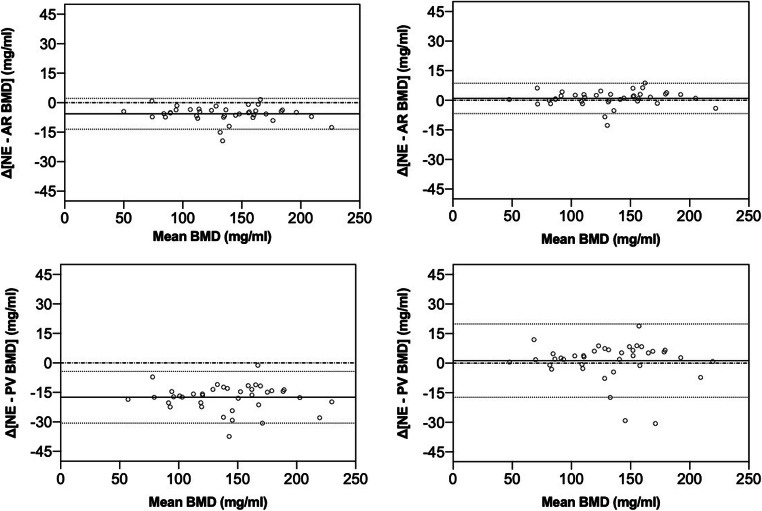
Bland-Altman plots show the means vs. the difference of the bone mineral density (BMD) values measured in contrast-enhanced and nonenhanced MDCT scans. Averaged (L1–L3) BMD values derived from contrast-enhanced scans differ significantly from nonenhanced (NE) scans (all *p* < .001). The effect of intravenous contrast agent is most notable in uncorrected portal-venous (PV) scans. After the automated correction with the anatomy-guided ANN, no significant difference is observed (all *p* > .05). Data points are observed data. The solid line indicates the mean difference. The dashed lines indicate the 95% limits of agreement. Upper row: arterial phase (AR) CT scans—not corrected and corrected. Lower row: portal-venous CT scans—not corrected and corrected

Averaged, uncorrected BMD values derived from contrast-enhanced CT images were significantly overestimated compared to NE MDCT scans (all *p* < .001). Uncorrected arterial (AR)-phase BMD values were approximately 4% higher (mean difference 5.68 mg/ml; 138.2 vs. 132.5 mg/ml), and PV-phase BMD values were approximately 13% higher (mean difference 17.5 mg/ml; 150.0 vs. 132.5 mg/ml). After the automated correction for contrast agent, no significant difference to NE MDCT scans was observed (AR, PV both *p* > .05; mean difference 0.94 mg/ml for AR; and 1.28 mg/ml for PV) (see Table 4 and Fig. 4 for respective mean differences). Accuracy comparison, calculated as the root-mean-square error, was 6.92 mg/ml for AR and 18.7 mg/ml for PV; root-mean-square error decreased to 3.98 mg/ml for AR and 9.45 mg/ml for PV, after the automated correction (Table 4).

**Table 4:** Accuracy comparison in the test set before and after the automated correction

	Corrected		Not corrected	
	AR	PV	AR	PV
RMSE (mg/ml)	3.98	9.45	6.92	18.7
Mean difference to NE BMD (mg/ml)	0.94	1.28	− 5.68	− 17.5

Note: Data are means

*RMSE* root-mean-square error, *BMD* bone mineral density, *AR* arterial, *PV* portal venous, *NE* nonenhanced

## Discussion

This study showed that an artificial neural network (ANN) can reliably detect the presence and phase of iodinated contrast agent in routine abdominal MDCT scans. The proposed ANN performed well both on the test set, as well as on the public dataset acquired with multiple different CT scanners, validating its generalizability and robustness to such a domain shift. As one possible application, we showed a significant improvement in opportunistic BMD assessment.

Three different ANN models were introduced and compared in this study. The random selection of 2D slices for contrast prediction seems to lack reproducibility. Using a full 3D scan, on the other hand, leads to memory constrains and higher number of parameters to be optimized decreases the performance. Thus, we propose an anatomy-guided 2D approach as the optimal model for an accurate contrast prediction.

Previous studies have stated that the effect of using intravenous contrast agent is negligible [30, 31]. However, the authors did not provide sufficient validation in terms of accuracy and precision, leaving such approaches questionable for individual BMD assessment [32]. Our data suggests that intravenous contrast administration is associated with a systemic bias in vertebral BMD measurements. This is in line with several studies reporting significant differences between enhanced and nonenhanced CT scans [12–15, 33]. Boutin and colleagues found a mean increase of 33 HU at L4 in the PV phase [12]. Pompe et al reported a mean difference of 19 HU at L1 between the NE and the PV phases. They stated that unadjusted CT scans may lead to an underdiagnosis of osteoporosis in 7–25% of patients [15]. In both studies, the mean difference was greatest in the PV phase. In our study, BMD values derived from PV scans revealed a mean difference of 17.5 mg/ml compared to those of NE scans. This equals almost half the BMD range between normal (BMD > 120 mg/ml) and osteoporosis (BMD < 80 mg/ml) defined by the American College of Radiology (ACR) [34].

Acu and colleagues suggested that scan delay time is a significant and quantifiable variable, due to the steady accumulation of contrast agent [35]. Our data supports this hypothesis, revealing that the increase in BMD values from AR to PV phase is statistically significant. This indicates that measurements are not only contrast dependent but also contrast phase dependent. This accuracy bias should not be neglected, especially in longitudinal studies with repeated measurements. Taken together, our findings argue for an adequate correction method. In our study, this was achieved through simple linear regression for each contrast phase. Regarding skeletal muscle measurements, changes are also known to occur and linear correction models for measurements have been proposed [12, 36]. This is important when using internal (in-body) calibration, as the assessed calibration tissue also experiences enhancement. Further studies will have to investigate on how to minimize other biases, such as patient diameter and patient positioning.

There are limitations to this retrospective study. As we focused on lumbar BMD measurements, we did not include CT scans which only cover the cervical spine for training. Further studies are needed to investigate the performance of the proposed framework in cervical spine examinations.

## Conclusion

In conclusion, the artificial neural network presented here works reliably in any given CT scan and could be integrated into various frameworks to complete the workflow of automated or semi-automated data extraction from routine contrast-enhanced CT images. We propose an anatomy-guided approach as the most accurate tool for automated contrast phase assessment. Besides the simple design and little computation-power requirements, the main advantage is the high diagnostic accuracy. This reduces false-negative results in osteoporosis screening.

## Abbreviations

2D: Two dimensional
3D: Three dimensional
ANN: Artificial neural network
AR: Arterial
BMD: Bone mineral density
NE: Nonenhanced
PV: Portal-venous
RMSE: Root-mean-square error

## Declarations

### Guarantor

The scientific guarantor of this publication is Jan S. Kirschke.

### Statistics and biometry

One of the authors has significant statistical expertise.

### Methodology

• retrospective

• diagnostic or prognostic study

• performed at one institution (with multicenter data)
